# A New Electric Horn for Motorists

**Published:** 1909-06-26

**Authors:** 


### Motoring Notes.

### A NEW ELECTRIC HORN FOR MOTORISTS.

The need of an electric horn which can be relied upon to keep in perfect working order for a considerable period and, at the same time, to give forth a loud, clear blast with a minimum current consumption has long been felt by motorists. Hitherto, the great trouble has been the sparking which takes place at the make and break contact points which in a short time renders the apparatus ineffective. This difficulty was occasionally overcome by using condensers, but the cure was almost as bad as the disease as the condensers required renewal more often than was desirable.

Another cause for anxiety to those motorists who had a liking for electric horns was the probability that the vibrating diaphragm would crack, perhaps during a violent effort to arouse a sleeping hay-cart driver. In the construction of the Aduil electric horn supplied by Messrs. Marples, Leach and Co., Limited, Aduil Buildings, Artillery Lane, E.C., these two drawbacks have been eliminated, without in any way interfering with the volume of sound. The spark resulting from the making and breaking contact is completely avoided by a special magnetic blow-out winding, whilst the vibrating diaphragm is constructed of a tough bronze alloy, the composition of which, it is stated, is known only to the makers. The sound is obtained on principles similar to those which are generally adopted in phonographs or gramophones.

The transformation of electric energy into sound is effected by means of an electro-magnetic contact-breaker which has the effect of setting up rapid oscillations of the diaphragm when the push (which is usually fitted on the steering-wheel) is depressed. It may be observed that the diaphragm makes approximately 320 oscillations per second, which practically corresponds to the lower E in music. The diaphragm hermetically closes the entrance to the interior of the apparatus, consequently no risk is incurred by using the horn in all sorts of weather.

The lowest pressure which these non-sparking horns are wound for is 8 volts, and the highest 220 volts. To meet the requirements of motorists the makers also supply horns to work at 4 volts, but although the sparking has been considerably reduced it has been found impossible entirely to overcome it without sacrificing much of the sound.

A few points about the current consumption of electric horns are worthy of note. An 8-volt horn takes less than 0.4 of an ampere, and a 12-volt horn 0.3 of an ampere. It will thus be seen that an accumulator having a capacity of 10-ampere hours is capable of producing over 10,000 blasts of a second's duration.

It will be observed from the above figures that the Aduil is a very considerable improvement on all electric horns hitherto offered to the motorist. Horns of this type are extremely useful when overtaking hooded vans, hay-carts, noisy farm wagons, etc., when the ordinary horn is ineffective, but up to now the drawbacks stated above have prevented their general use. It should be stated here that this improved type of horn is placed on the market at a very reasonable figure, and may be purchased in the straight or twisted pattern.

